# A dual doping nonvolatile reconfigurable FET

**DOI:** 10.1038/s41598-023-32930-9

**Published:** 2023-04-06

**Authors:** Xiaoshi Jin, Shouqiang Zhang, Xi Liu

**Affiliations:** School of Information Science and Engineering, Shenyang University of Technology, Shenyang, 110870 China

**Keywords:** Applied physics, Nanoscale devices, Electrical and electronic engineering, Engineering

## Abstract

In this work, we propose a dual doping based nonvolatile reconfigurable field effect transistor with source/drain (S/D) charge storage layers (DDN R-FET). It introduces nonvolatile charge storage layers on both source and drain sides as a floating program gate (FPG) instead of a program gate (PG) that needs independent power supply. The stored charges in the FPG are programmed by the control gate (CG). Therefore, the proposed DDN R-FET essentially requires only one independently powered gate to complete the reconfigurable operation. Moreover, by adjusting the charge stored in the FPGs, the CG can regulate the equivalent voltage in the FPG, which can promote the on-state current and reduce the generation of reversely biased leakage current at the same time. The physical mechanism has also been analyzed in details.

## Introduction

As the scale of CMOS reaches the physical limit in the next decade, improvements such as enhance the function of single electronic device are needed to achieve more complex systems with a lower number of devices, thereafter, higher flexibility of hardware and more simplified technology implementation can be realized and the value per building block of integrated circuits can be increased. Recently, the polarity controllable field effect transistors, or reconfigurable field effect transistor (RFET) is proposed. As a single device, it can be configured as n-type or p-type FET by resetting the voltage applied on the PG during operation^1–3^. Thereafter, RFET is possible to offer an advantage in programmable logic arrays and realizes various logic gates with fewer transistors than conventional CMOS technology^4–8^. Since RFET blocks the source and drain to form ohmic contact by forming Schottky barrier in the source region, and it is turned on through tunneling effect, the forward current is smaller than that of mainstream CMOS technology. For increasing the forward current, the program gate has to be biased at a higher voltage, which causes a increasing potential difference between the CG and the PG when the CG is at low potential or reverse bias, resulting in the generation of leakage and the increase of power consumption, especially when the distance between the PG and the CG is reduced to deep nanoscale. A dual doping source/drain RFET (DD R-FET) which is with dual doping source and drain located next to each other is proposed^9,10^, it significantly reduces the required voltage of the PG and the on-state current is also improved. However, compared with single gate FETs, the extra PG of RFET increases the complexity and difficulty of metal interconnection. Since the PG is always works at high voltage level, strong band bending induced tunneling effect will induced a large amount of leakage current especially when the gate electrode is reversely biased. This effect is particularly significant for highly integrated RFET. In this paper, we propose a dual doping nonvolatile reconfigurable field effect transistor with source/drain (S/D) FPGs (DDN R-FET). Different from the conventional RFET, it introduces nonvolatile charge storage layers as FPGs instead of PGs that needs independent power supply. The stored charge in the FPG can be programmed by applying a high voltage to the CG. Therefore, the proposed DDN R-FET essentially requires only one independently powered gate to complete the reconfigurable operation. Moreover, the CG can regulate the equivalent voltage in the FPG. It can promote the on-state current and reduce the reversely biased leakage current at the same time. The physical mechanism has also been analyzed in details.

## Structure and parameters

**Figure 1:**
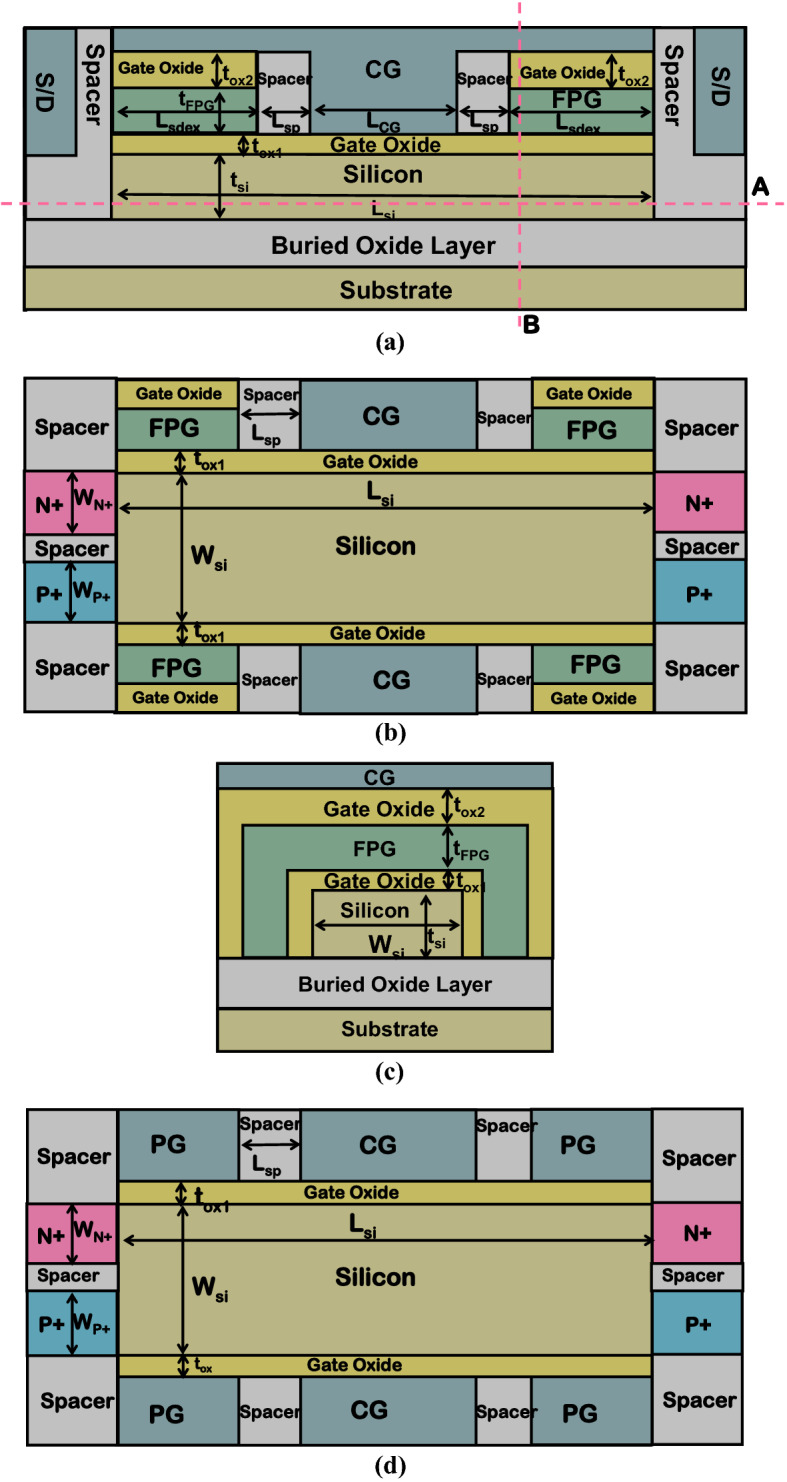
(**a**) A cross view of DDN R-FET. (**b**) The cross view of DDN R-FET along cutline A in Fig. 1a. (**c**) The cross view of DDN R-FET along cutline B in Fig. 1a. (**d**) A schematic view of DD R-FET.

Figure 1a is a cross view of the proposed DDN R-FET, Fig. 1b and c are cross views obtained along the cutline A and cutline B in Fig. 1a, respectively. Figure 1d is a schematic view of DD R-FET. L_si_ is the length of silicon body from the source electrode to the drain electrode. L_CG_ is the bottom length of the CG. L_sdex_ is the length of the floating programmable gate (FPG). L_sp_ is the length of the spacer between the CG and the FPG. t_si_ is the thickness of the silicon body, t_ox1_ is the thickness of the gate oxide on the top of the silicon body, t_ox2_ is the thickness of the gate oxide between the CG and the FPG. t_FPG_ is the thickness of the FPG. W_si_ is the width of the silicon body. N_D_ and N_A_ are the donor and acceptor concentrations of the N + and P + source /drain regions, respectively. W_N+_ and W_P+_ are the widths of the N + and P + source/drain regions, respectively. The performance and the comparisons between the proposed DDN R-FET and the DD R-FET are verified by device simulation using SILVACO tools^11^. Physical models such as Fermi distribution model, CVT mobility model, auger recombination model, band gap narrowing model and a standard band to band tunneling model are all turned on. Considering that the most advanced lithography technology can achieve a gate length of about 5 ~ 10 nm, and the thickness of the gate oxide layer can be reduced to about 1 nm, the L_CG_ is set to be 10 nm and L_sdex_ is set to be 5 nm, t_ox1_ is set to be 2 nm. Nonvolatile charge storage layers as FPGs instead of PGs that needs independent power supply. The stored charge in the FPG can be programmed by applying a high voltage to the CG. Therefore, the proposed DDN R-FET essentially requires only one independently powered gate to complete the reconfigurable operation. To realize the operation of writing charge to FPG, t_ox2_ should be thicker than t_ox1_ so that the charge can be written more easily, and t_ox2_ is set to be 3 nm. In order to strengthen the control of the gate on the channel potential, t_si_ should not be too thick, so here we set t_si_ to be 5 nm. In order to avoid the increase of series resistance, L_sp_ should be as short as possible, so we set L_sp_ to be 5 nm. In order to ensure the objectivity of the comparison, we have adopted the most consistent parameter settings for DD R-FET. Moreover, the CG can regulate the equivalent voltage in the FPG. It can promote the on-state current and reduce the reversely biased leakage current at the same time. The physical mechanism has also been analyzed in details.

## Results and discussion

**Figure 2:**
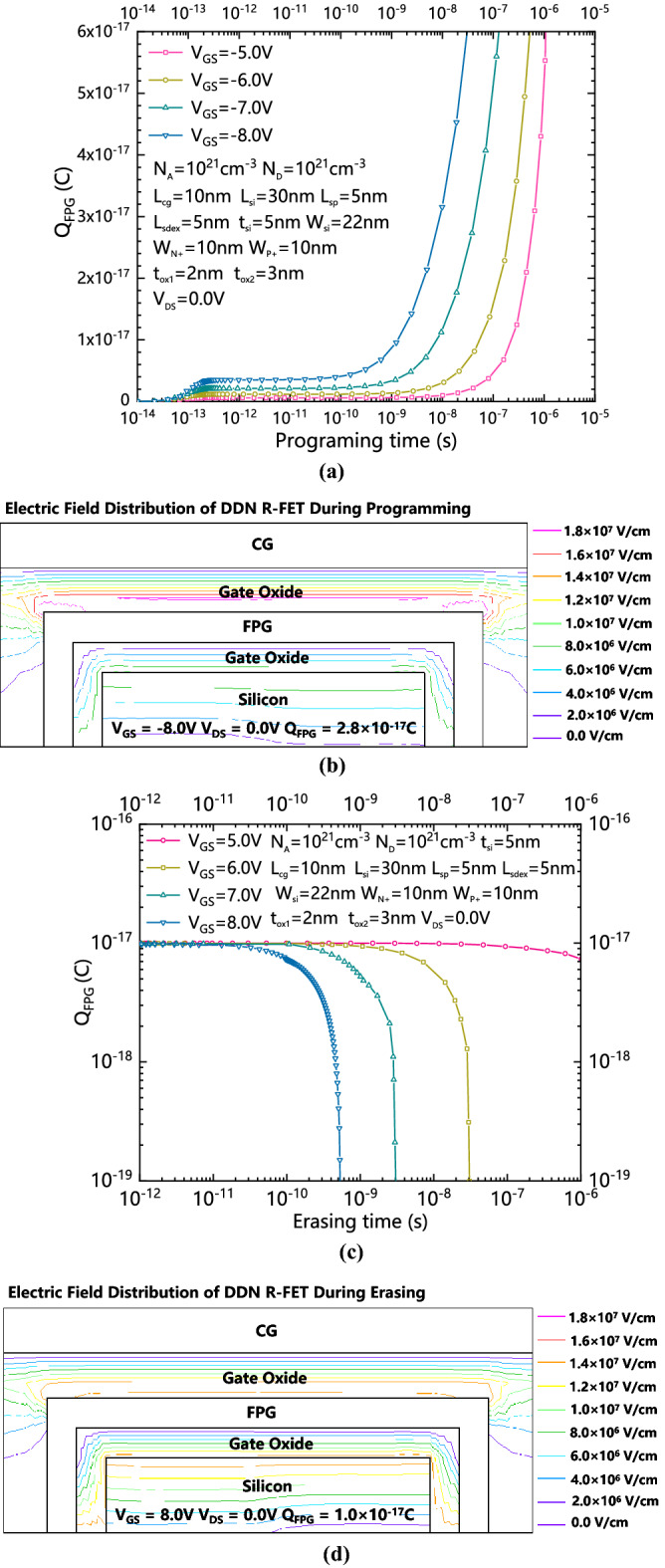
(**a**) The dependence between Q_FPG_ and programming time under different V_GS_s. (**b**) The electric field distribution of DDN R-FET during programming with a − 8 V V_GS_. (**c**) The dependence between Q_FPG_ and erasing time with different V_GS_ s with an initial Q_FPG_. (**d**) The electric field distribution of DDN R-FET during erasing with an 8 V V_GS_.

Figure 2a shows the dependence between Q_FPG_ and programming time with different V_GS_ s. Figure 2b shows the electric field distribution of DDN R-FET during programming with a −8 V V_GS_. Figure 2c shows the dependence between Q_FPG_ and erasing time with different V_GS_ s with an initial Q_FPG_. Figure 2d shows the electric field distribution of DDN R-FET during erasing with an 8 V V_GS._ When FPG is being programmed, the source/drain electrodes are grounded, and the gate electrode is applied to be a relatively large voltage. Q_FPG_ is roughly proportional to the programming time, and the programming time can be shorted by applying a larger negative V_GS_.

In order to produce significant gate oxide tunnel effect during programming or erasing operation, the electric field intensity applied to the gate oxide layers is typically greater than 10^7^ V/cm. After programming by a negative V_GS_, positive charges are stored in the FPG and the proposed DDN R-FET can work in N-mode. The positive Q_FPG_ can be erased by applying a large positive V_GS_. The erasing time is inversely proportional to the V_GS_.

**Figure 3:**
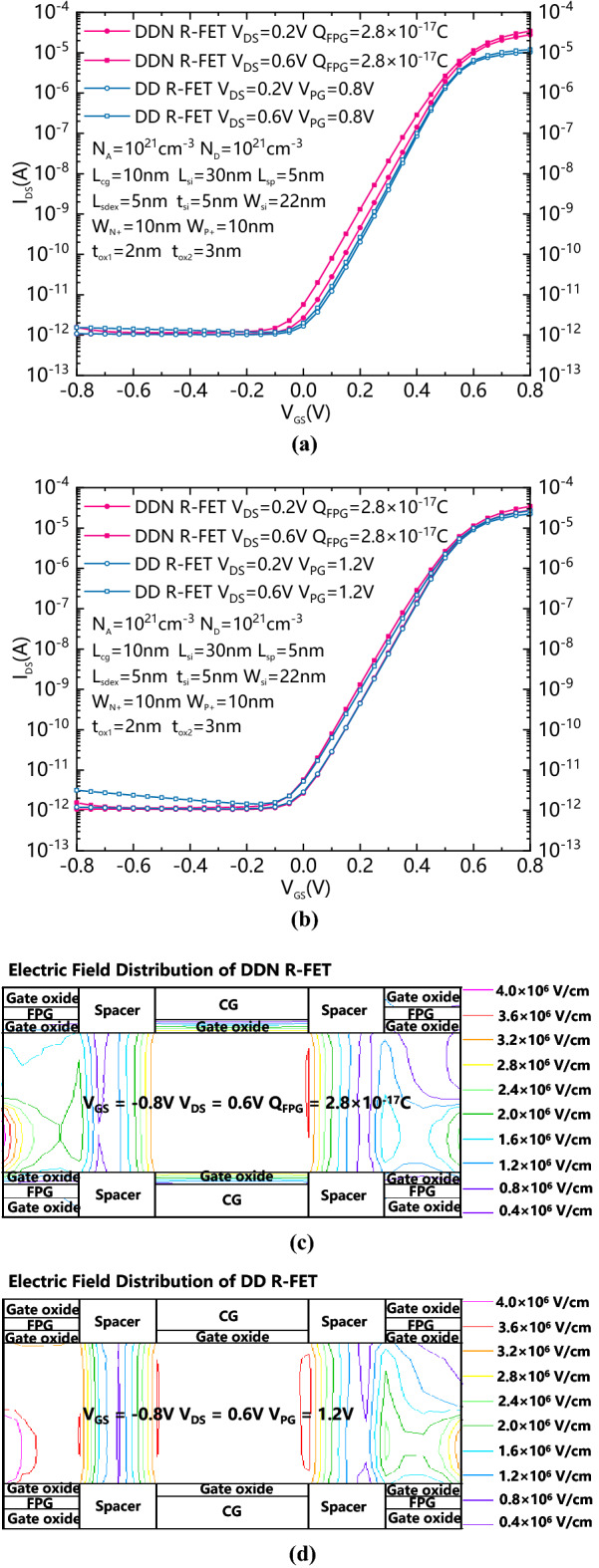
(**a**) Comparison of transfer characteristic between DDN R-FET and the DD R-FET with V_PG_ equals to 0.8 V. (**b**) Comparison of transfer characteristic between DDN R-FET and the DD R-FET with V_PG_ equals to 1.2 V. (**c**) Electric Field distribution of DDN R-FET in reversely biased n-mode state. (**d**) Electric Field distribution of DD R-FET in reversely biased n-mode state.

Figure 3a is the comparison of transfer characteristic between DDN R-FET and the DD R-FET with V_PG_ equals to 0.8 V. Figure 3b is the comparison of transfer characteristic between DDN R-FET and the DD R-FET with V_PG_ equals to 1.2 V. When the V_PG_ of DD R-FET equals to 0.8 V, the forward conduction current is smaller than that of DDN R-FET, while the leakage current of DD R-FET is almost the same with that of DDN R-FET. When the V_PG_ of DD R-FET is equal to 1.2 V, compared with DDN R-FET, the forward conduction current is similar to that of DDN R-FET, while the reverse leakage current also increases and is larger than that of DDN R-FET. Figure 3c shows the electric field distribution of DDN R-FET in reversely biased n-mode state with Q_FPG_ equals to 2.8 × 10^−17^C. Figure 3d shows the electric field distribution of DD R-FET in reversely biased n-mode state with V_PG_ equals to 1.2 V. The electric field intensity of DDN R-FET is smaller than that of DD R-FET. Therefore, for DD R-FET, a fixed V_PG_ is not conducive to increase the forward current and restrain the band to band tunneling induced reverse leakage current at the same time.

**Figure 4:**
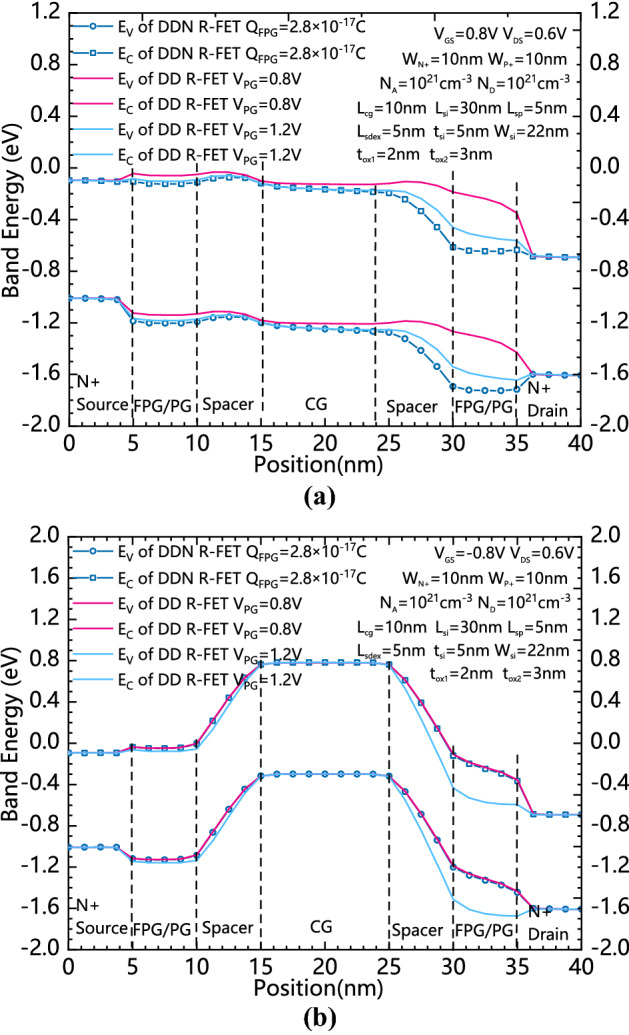
(**a**) Comparison of Energy band diagram between DDN R-FET and DD R-FET with positively charged FPGs and forwardly biased CG. (**b**) Comparison of Energy band diagram between DDN R-FET and DD R-FET with positively charged FPGs and reversely biased CG.

Figure 4a and b show comparison of Energy band diagram between positively charged DDN R-FET and positively programmed DD R-FET with forwardly biased CG and reversely biased CG, respectively. Due to that the effective voltage in the FPG is determined by both the controlled gate voltage and the amount of charge stored inside it, the positively biased CG and the positively charged FPG of the DDN R-FET provide an enhanced flow path for the electrons in the conduction band. As Fig. 4a shows, for DD R-FET, if conduction path similar to DDN R-FET is obtained, the voltage of PG should be increased to more than 1.2 V. However as shown in Fig. 4b, when the CG is reversely biased, the effective voltage of the FPG is pulled down due to coupling effect between the CG and FPG. Therefore, the energy band bending on both sides of the source and drain is appropriately reduced, which can inhibit the reversely biased leakage current, while a increased VPG induced stronger band bending in the reversely biased state, which leads to an increased band to band tunneling leakage current. Therefore, as Fig. 3a and Fig. 3b shows, the forward biased on-state current of DDN R-FET and reversely biased leakage current can be improved at the same time comparing to DD R-FET.

**Figure 5:**
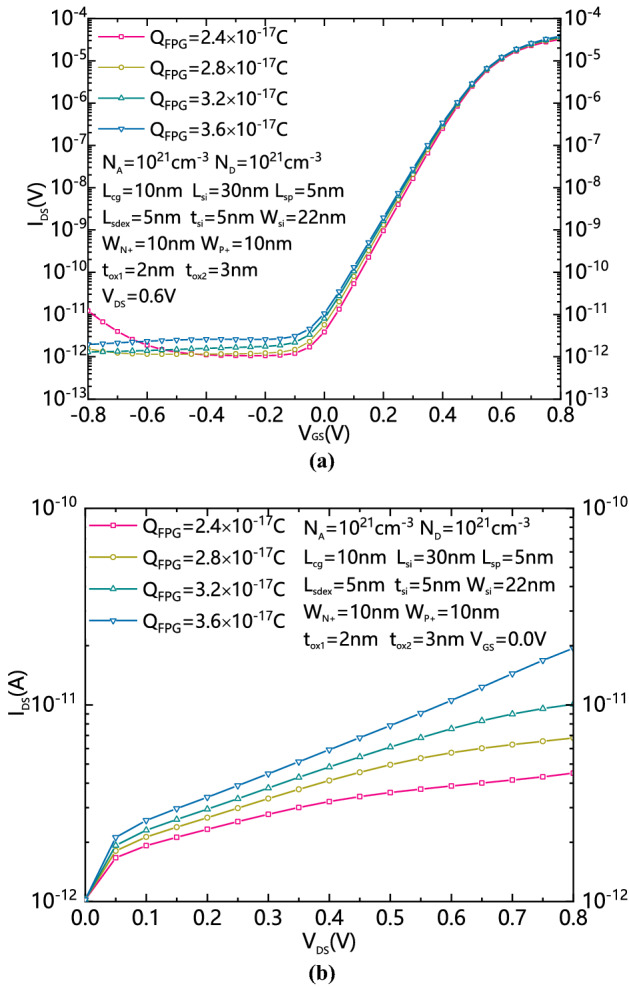
(**a**) Comparison of transfer characteristic of DDN R-FET with different QFPGs and VDS equals to 0.6 V. (**b**) Comparison of standby leakage current of DDN R-FET with different QFPGs and VDSs.

Figure 5a shows the comparisons of transfer characteristic of DDN R-FET with different Q_FPG_s and V_DS_ equals to 0.6 V. Figure 5b shows the comparison of leakage current at 0 V of V_GS_ with different Q_FPG_s and V_DS_s. The amount of Q_FPG_ has almost no effect on the transfer characteristic. A larger Q_FPG_ will leads to the increase of leakage current, just as the PG of DD R-FET is applied to a high voltage which induced strong band bending. Therefore, the amount of charge stored in the FPG should be controlled within a reasonable range. The recommended value of Q_FPG_ is about 2.8 × 10^−17^C for the proposed DDN R-FET in this work.

**Figure 6:**
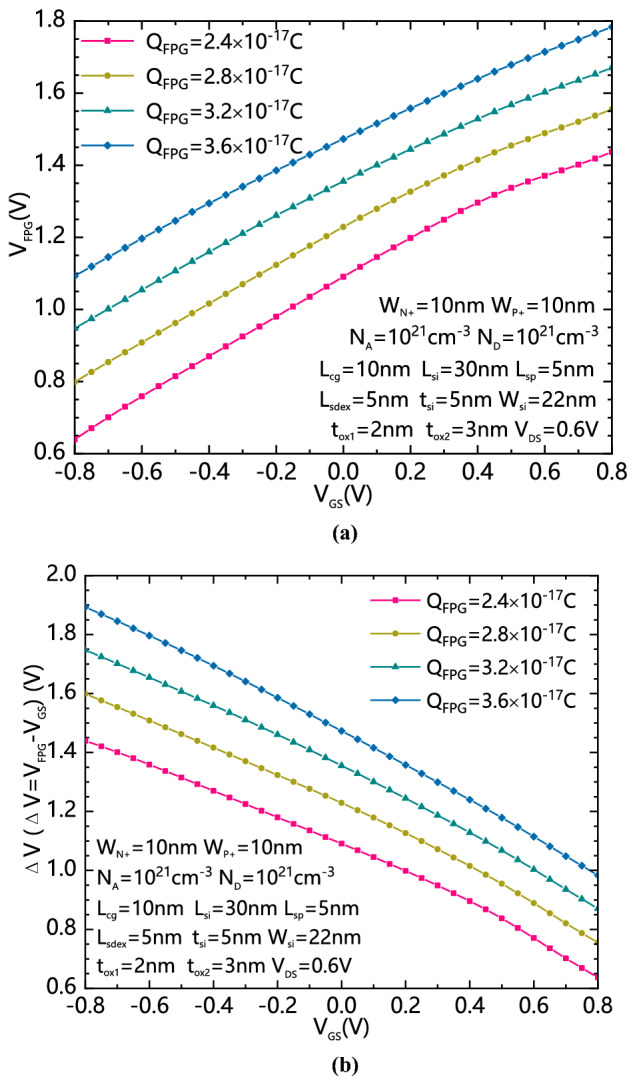
(**a**) The variation of the VFPG with different QFPGs and VGSs; (**b**) the variation of △V with different QFPGs andVGSs.

Figure 6a shows the variation of the effective voltage of the floating grogram gate V_FPG_ with different Q_FPG_s and V_GS_s. Figure 6b shows the variation of the voltage difference between FPG and CG △V with different V_GS_s. V_FPG_ is generally proportional to the increasing of the Q_FPG_s and the increasing of V_GS_s, thereafter, by controlled the amount of Q_FPG_ to be within a reasonable range, V_FPG_ can achieve a sufficiently high equivalent voltage when CG is forwardly biased and a sufficiently low voltage when CG is reversely biased. △V is generally reduced with the reducing of the amount of Q_FPG_. By properly reducing Q_FPG_, the △V can be reduced, so as to reduce the energy band bending and reduce the generation of leakage current.

**Figure 7:**
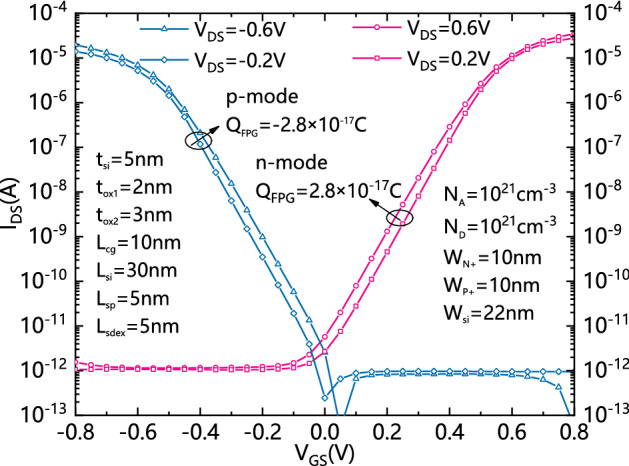
Transfer characteristic of DDN R-FET in both n-mode and p-mode with positively charged and negatively charged FPGs.

Figure 7 shows transfer characteristics in both n-mode and p-mode of the proposed DDN R-FET. It can be seen that it works in n-mode and p-mode by charging FPG positively and negatively, respectively. The reversely biased leakage current is reduced with the increasing of the absolute value of V_GS_. Due to the difference of mobility between electrons and holes, the device does not present symmetric current.

**Figure 8:**
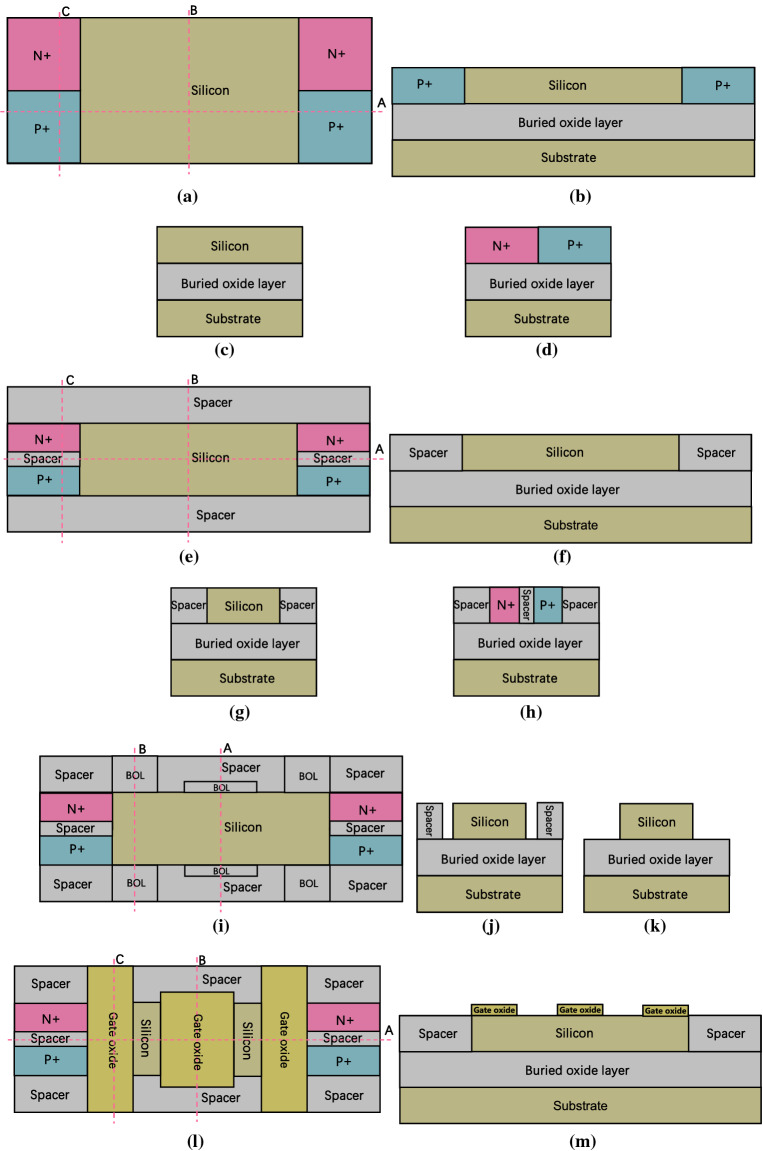

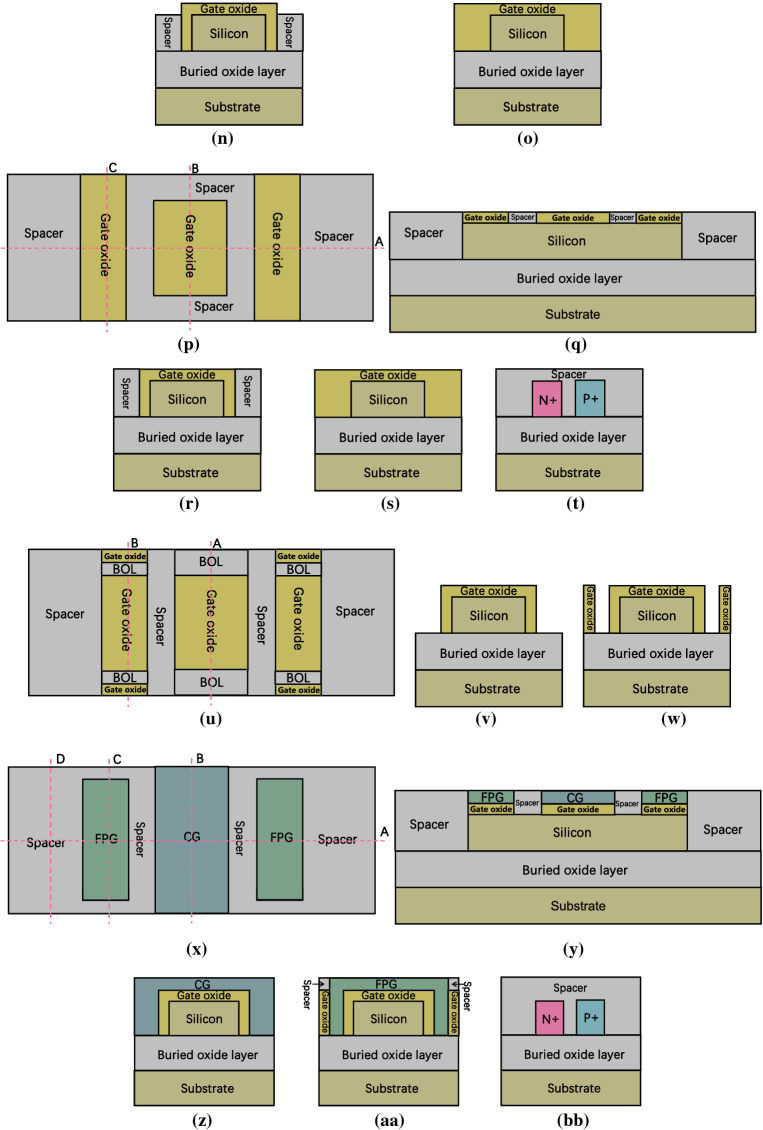

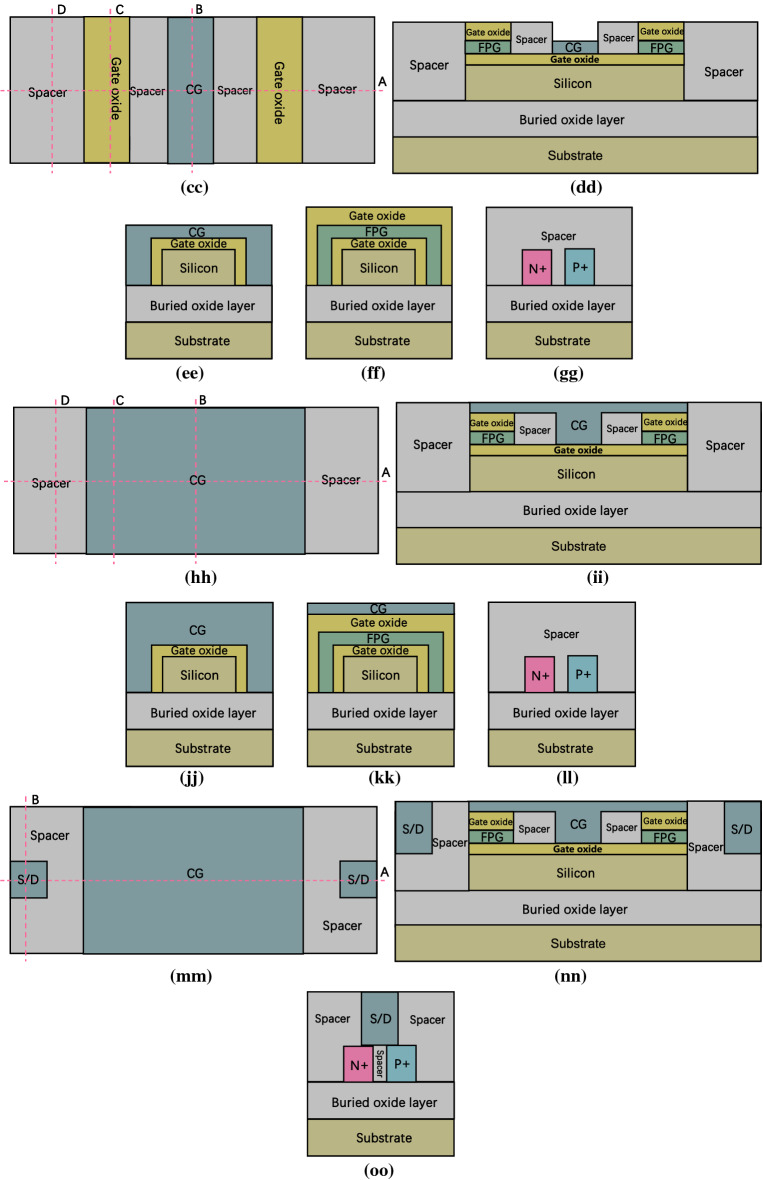
(**a**) top view of step 1, (**b**) cross view of Fig. 8a along cutline A, (**c**) cross view of Fig. 8a along cutline B, (**d**) cross view of Fig. 8a along cutline C, (**e**) top view of step 2, (**f**) cross view of Fig. 8e along cutline A, (**g**) cross view of Fig. 8e along cutline B, (**h**) cross view of Fig. 8e along cutline C, (**i**) top view of step 3, (**j**) cross view of Fig. 8i along cutline A, (**k**) cross view of Fig. 8i along cutline B, (**l**) top view of step 4, (**m**) cross view of Fig. 8l along cutline A, (**n**) cross view of Fig. 8l along cutline B, (**o**) cross view of Fig. 8l along cutline C, (**p**) top view of step 5, (**q**) cross view of Fig. 8p along cutline A, (**r**) cross view of Fig. 8p along cutline B, (**s**) cross view of Fig. 8p along cutline C, (**t**) cross view of Fig. 8p along cutline D, (**u**) top view of step 6, (**v**) cross view of Fig. 8**u** along cutline A, (**w**) cross view of Fig. 8u along cutline B, (**x**) top view of step 7, (**y**) cross view of Fig. 8x along cutline A, (**z**) cross view of Fig. 8x along cutline B, (**aa**) cross view of Fig. 8x along cutline C, (**bb**) cross view of Fig. 8x along cutline D, (**cc**) top view of step 8, (**dd**) cross view of Fig. 8cc along cutline A, (**ee**) cross view of Fig. 8cc along cutline B, (**ff**) cross view of Fig. 8cc along cutline C, (**gg**) cross view of Fig. 8cc along cutline D, (**hh**) top view of step 9, (**ii**) cross view of Fig. 8hh along cutline A, (**jj**) cross view of Fig. 8hh along cutline B, (**kk**) cross view of Fig. 8hh along cutline C, (**ll**) cross view of Fig. 8hh along cutline D, (**mm**) top view of step 10, (**nn**) cross view of Fig. 8mm along cutline A, (**oo**) cross view of Fig. 8mm along cutline B.

Figure 8 show a brief fabrication flow of the proposed DDN R-FET. As shown in Fig. 8a to Fig. 8d, prepare a SOI wafer, the bottom of the SOI wafer is the silicon substrate. The top of the SOI wafer is the silicon film. The buried oxide layer (BOL) is sandwiched between them. Dope both the left and right side of the silicon film by ion implantation process twice to form N + and P + regions. As shown in Fig. 8e–h, remove the front and back part of the silicon film and part of the doped region between N + and P + region above the SOI wafer through the photolithography and etching processes, then deposit insulator material and flatten the surface till expose the silicon film through chemical mechanical polishing (CMP) process to initially form the spacer region. Then the N + and P + regions are isolated by the spacer. As shown in Fig. 8i–k, remove parts of the spacer in front and back of the silicon film till that the BOL is exposed through photolithography and etching processes. Then some space is reserved for some parts of gate oxide layer and FPG. As shown in Fig. 8l–o, deposit high-k dielectric material and flatten the surface through CMP process to adjust the thickness of the gate oxide layer, then remove parts of the high k dielectric material to expose parts of silicon, parts of spacer, the N + and the P + regions, then the gate oxide layer for both CG and FPG is initially formed. As shown in Fig. 8p–t, deposit insulator material and flatten the surface to expose the gate oxide layer through CMP process to further form the spacer regions. As shown in Fig. 8u–w, remove parts of high k dielectric regions till expose the BOL to reserve some space for some parts of the FPG and CG through photolithography and etching processes. As shown in Fig. 8x–bb, deposit polysilicon or metal material, then flatten the surface through CMP process to adjust the thickness of the metal layer for the formation of CG and FPG, then remove parts of the polysilicon metal layer through photolithography and etching processes to initially form CG and FPG. After that, deposit insulator material and flatten the surface to expose CG and FPG to further form the spacer regions. As shown in Fig. 8cc–gg, remove some of the spacer region above the gate oxide region to expose the gate oxide in front and back of the FPG, then deposit the high-k dielectric material again and flatten the surface through CMP process, then remove some parts of the high k dielectric material through photolithography and etching processes to expose the surface of the spacer regions between the FPG and CG, then deposit insulator material again and flatten the surface through CMP process to expose the second layer of the gate oxide, then remove the high-k dielectric material above the CG through photolithography and etching processes, thereafter, the second gate oxide layer is formed. As shown in Fig. 8hh–ll, deposit polysilicon or metal material, flatten the surface through CMP process, then remove parts of the metal layer on both the source and drain sides through photolithography and etching processes to expose the surface of spacer regions, through the process above to further form CG, then deposit the insulator material again and flatten the surface to expose the surface of the spacer regions through CMP process. As shown in Fig. 8mm–oo, remove parts of the spacer region on both source and drain sides to expose the N + and P + regions. Then deposit metal material and flatten the surface to expose the surface of CG and spacer regions through CMP process to form the source and drain electrodes.

## Conclusion

In this paper, we propose a novel single gate controlled DDN R-FET. Different from the DD R-FET, it is with only one independently powered gate, which can do the reconfigurable operation and the control of the transistor to be switched between the on and off states. Moreover, by storing charge in the FPG within a reasonable range, the CG can regulate the equivalent voltage in the FPG, which can effectively reduce the generation of reverse leakage current. Therefore, the proposed DDN R-FET not only simplifies the structure, but also brings nonvolatile function and improves the device performance, comparing to conventional DD R-FET.

## Data Availability

The datasets used and/or analyzed during the current study available from the corresponding author on reasonable request.
